# Telomeric ORFS in *Candida albicans*: Does Mediator Tail Wag the Yeast?

**DOI:** 10.1371/journal.ppat.1004614

**Published:** 2015-02-12

**Authors:** Derek J. Sullivan, Judith Berman, Lawrence C. Myers, Gary P. Moran

**Affiliations:** 1 Division of Oral Biosciences, Dublin Dental School and Hospital, University of Dublin, Trinity College Dublin, Dublin, Ireland; 2 Department of Molecular Microbiology & Biotechnology, George S. Wise Faculty of Life Sciences, Tel Aviv University, Ramat Aviv, Israel; 3 Department of Biochemistry, Geisel School of Medicine at Dartmouth, Hanover, New Hampshire, United States of America; McGill University, CANADA

## Introduction

Recent studies of fungal genomes have shown that subtelomeric regions of chromosomes are areas of rapid evolution that facilitate adaptation to novel niches [1]. Several years ago, analysis of the genome of the human pathogenic yeast *Candida albicans* revealed the presence of a large family of telomeric orfs (*TLO* genes) [2]. The function of this gene family remained an enigma in *C*. *albicans* genetics for many years; however, recent studies have revealed that the *TLO* genes encode a subunit of the Mediator complex with roles in transcriptional regulation [3,4]. This gene family expansion is unique to *C*. *albicans*, the species responsible for the majority of human yeast infections and the species that is most commonly recovered as a human commensal. If selective pressures in the host have driven this expansion, it is likely that this gene family somehow contributes to the success of *C*. *albicans* as a commensal and opportunistic pathogen. To support this hypothesis, it was first necessary to determine the exact function of Tlo proteins in *C*. *albicans*. Armed with this knowledge, investigators are now beginning to understand how possession of multiple copies of *TLO* could contribute to the virulence properties of *C*. *albicans*.

## What Are the Tlo Proteins?

The earliest reference to a *TLO* gene was made by Kaiser et al. [5] who identified *CTA2* (now *TLOα3*) because it encoded transcriptional activating activity in a yeast one-hybrid screen. Goodwin and Poulter [6] later noted that sequences homologous to this ORF were commonly found at telomeres in *C*. *albicans*, indicating that these genes were widely dispersed. Subsequently, annotation of the *C*. *albicans* genome revealed 14 *TLO* family members in strain SC5314 (13 telomeric; one centromeric) [2]. The large expansion of the *TLO* gene family is, however, unique to *C*. *albicans* (there are two copies in *C*. *dubliniensis* and only one in the sequenced genomes of other *Candida* species). In silico analysis of these sequences by Bourbon et al. [7] suggested that the *TLO* genes encode proteins with a domain similar to the *Saccharomyces cerevisiae* Med2 protein, a component of Mediator.

**Fig 1:**
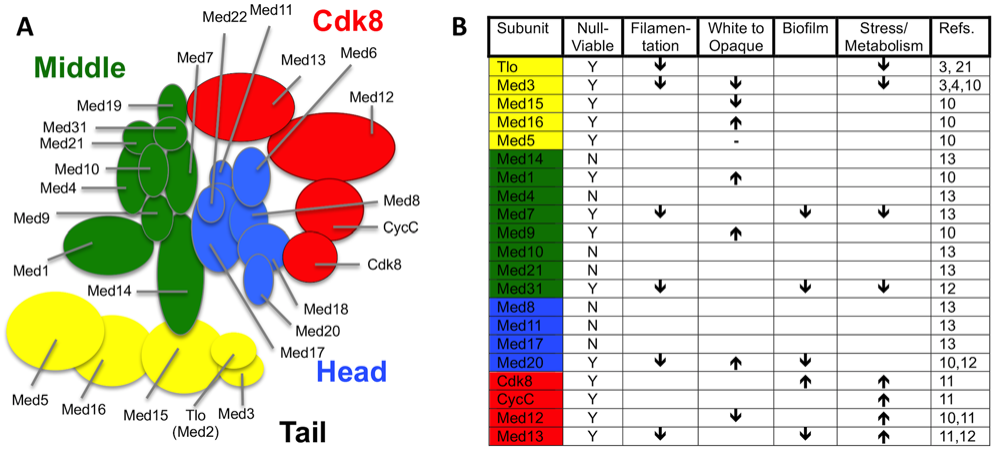
Current knowledge on structure and function of *C*. *albicans* and *C*. *dubliniensis* Mediator. (A) Predicted structure of *C*. *albicans* (and *C*. *dubliniensis*) Mediator based on structural analysis of *S*. *cerevisiae* complex [25]. Biochemical analyses of Mediator Tail subunits from *C*. *albicans* [3,10] and *C*. *dubliniensis* [3] supports the proposed structure of this module in the pathogens. Biochemical studies provide direct evidence that Tloβ2, Tloα3, Tloα9, Tloα12, and Tloα34 are mutually exclusive Med2 orthologs of the *C*. *albicans* Mediator complex, while not excluding other expressed Tlo paralogs [4]. Additional biochemical studies show most Mediator in *C*. *dubliniensis* incorporates the Tlo1 subunit, but does not rule out the possibility that Tlo2 could associate with the complex under conditions in which its expression is increased [3]. (B) Summary of virulence related phenotypes associated with *C*. *albicans* and *C*. *dubliniensis* null mutants of genes encoding individual Mediator subunits. “Filamentation” includes defects in the yeast to hyphae transition. The “White to Opaque” arrows refer specifically to the white to opaque cell phenotypic switching frequency, including also the opaque to white cell switch frequency. “Biofilm” defects specifically refer to the ability to form a structure on a hard solid (i.e., plastic) support. “Stress/Metabolism” is a broad catchall that refers to the cell’s ability to remodel its internal metabolic wiring to respond to environmental stresses such as changes in carbon source, as well as oxidative and heat stress. Detailed information on each of these phenotypes can be found briefly within the body of the text, and in more detail in the references cited.

Zhang et al. [4] carried out the first purifications of Mediator complex from *C*. *albicans* and were able to identify Tlo proteins as stochiometric components of Mediator. Mediator is a large multisubunit complex that plays a primary role in facilitating physical and functional interactions between DNA-bound transcription factors and RNA polymerase II (Pol II) to activate transcription [7–10]. Recent analysis of Mediator functions in *C*. *albicans* have shown that this complex plays an important role in regulating many virulence-associated traits such as filamentous growth, white—opaque switching, stress responses, biofilm formation, and phagocyte interactions (Fig. 1) [10–14]. Mediator in fungi has 25 subunits organised into four distinct modules: a head module that interacts with Pol II, a regulatory middle module, and a tail module that includes Med2, Med3, and Med15, and which may play a direct role in transcriptional regulation [8]. A fourth variably associated Cdk8 module both negatively and positively regulates transcription [4,15]. Mediator purified from a *med3*Δ mutant lacked the Tlo subunit, strongly suggesting that Tlo proteins are Mediator tail subunits anchored to the complex via Med3. However, *C*. *albicans* cells also contained an excess of non-Mediator—associated Tlo protein, with this “free Tlo” form estimated to be at least 10-fold more abundant than the Mediator-associated form [4]. Whether the free-Tlo population carries out functions distinct from those of the Mediator bound form, or whether it acts as a reservoir of Tlo protein that can interchange with the Mediator bound subunits remains to be explored. In addition to the expansion of the Tlo orthologs of Med2 in *C*. *albicans* and *C*. *dubliniensis*, there are several other species and specific circumstances in which the copy number of a Mediator subunit exceeds the norm [7]. Intriguingly, the human fungal pathogen *Candida glabrata* has two paralogs of the Med15 Tail module subunit, which have both overlapping and non-overlapping functionality [16]. An increase in copy number of the human Mediator subunit Cdk8, which is accompanied by increased expression, is found in 70% of colorectal cancer samples and is significantly correlated with increased colon cancer—specific mortality [17].

## How Did the *TLO* Family Evolve in *C*. *albicans*?

Analysis of the synteny of the *TLO* genes in *Candida* species suggests that *C*. *albicans TLO2* corresponds to the ancestral locus, as a *TLO2* orthologue is present in the same subtelomeric locus in all related *Candida* species (i.e., *C*. *tropicalis*, *C*. *parapsilosis*, and *C*. *dubliniensis*). Translocation of an ancestral Med2 gene to this telomeric locus appears to have occurred early in the evolution of the clade, remaining stable until the emergence of the closely related species *C*. *dubliniensis* and *C*. *albicans*. *C*. *dubliniensis* harbors a second *TLO* gene internally on chromosome 7, whereas *C*. *albicans* underwent a massive expansion in *TLO* copy number, probably facilitated by subtelomeric recombination. Further diversification of the *C*. *albicans TLO* gene family was likely driven by retrotransposon activity, as three distinct subfamilies of *TLO*s (α, β, and γ) can be identified based on the presence of retrotransposon LTR sequences within the 3′-half of the gene [18]. Studies have shown that these *TLO* gene subfamilies are variably expressed in in vitro—grown cells, with the *TLO*α and *TLO*β genes and their encoded proteins expressed at higher levels than the *TLO*γ genes [18]. Concomitant with this, biochemical studies have directly shown that Tloβ2, Tloα3, Tloα9, Tloα12, and Tloα34 can be copurified with Mediator in vitro [4].

## Mediator: Monolithic or Multifaceted?

Large multisubunit coregulatory complexes, like Mediator, were once thought of as monolithic intermediaries in gene regulation, but the discovery of the Tlos as Mediator subunits are part of an emerging view of these complexes as dynamic entities whose functionality can be regulated. The evidence presented by Zhang et al. [4] suggests that Tlo proteins encode interchangeable Med2-like subunits of the Mediator tail. Most fungi appear to have one copy of Med2, which raises the question, what advantage could the expression of multiple Med2 subunits confer on *C*. *albicans*? In *S*. *cerevisiae*, Med2, as well as the Mediator tail in general, interacts with transcriptional activators to facilitate the transcription of highly inducible genes [8]. The Mediator tail is thought to be especially important for the regulation of stress responses and nutrient acquisition in *S*. *cerevisiae* [9]. These characteristics are also important in pathogenic fungi. However, why this function was amplified to such a great degree in *C*. *albicans* and whether this is connected to stress survival and nutrient status is not known.

Perhaps the amplification and divergence of the Med2 tail subunit facilitated the emergence of Mediator variants with specific regulatory functions. Transcriptional control of the *C*. *albicans* Tlos in response to pathways that impact pathogenesis [19,20] suggests that regulation of the Tlo pool could influence virulence gene expression. Testing such a hypothesis in *C*. *albicans* using traditional reverse genetic approaches on the 14 diploid *TLO* genes is a daunting challenge. Fortunately, the closely related species *C*. *dubliniensis* possesses only two *TLO* gene copies [21]. *C*. *dubliniensis* shares many characteristics with *C*. *albicans* (including the capacity to produce hyphae), but is responsible for far fewer infections and is generally less pathogenic in animal models of infection [22]. Haran et al. [3] deleted both *TLO* genes in *C*. *dubliniensis* and found virulence-associated phenotypes such as an inability to form true hyphae, increased susceptibility to oxidative stress, and a reduced capacity to assimilate alternative carbon sources. Transcript profiling indicated defective induction of filament-specific genes and regulators, e.g., *UME6*. Interestingly, many filament-specific genes were induced in *tloΔ* null mutants, but to a much lower level than in the wild-type parental strain, implying that the Tlo protein is required for full induction of the filament-specific transcriptional response. The *tlo*Δ null mutant also exhibited reduced expression of stress response and galactose utilization genes, indicating a general defect in inducible transcriptional responses. Expression data also suggested that Tlos may have repressor functions, as many starvation responses (gluconeogenesis, glyoxylate cycle, amino acid catabolism) were induced in the mutant [3].

*C*. *dubliniensis* yeast cells express Tlo1 at a level comparable to other Mediator subunits. However, expression of Tlo2 is far lower [3]. Deletion of *TLO1* appeared to have stronger effects on filamentous growth and growth in galactose, consistent with the near complete restoration by *TLO1* of those phenotypes in the *tlo∆* null. Restoration of *TLO1* or *TLO2* expression, at a level comparable to native *TLO1*, in the *tlo*Δ null mutant was found to restore the expression of overlapping and distinct sets of genes. If each of two *C*. *dubliniensis MED2* orthologs exhibits diversity, does each of the 14 *C*. *albicans TLO/MED2* orthologs also affect expression differently? The answer to this question awaits the results of studies currently underway to analyse the roles of individual *CaTLO* genes.

Evidence to date suggests that the *C*. *albicans TLO* gene family is subject to several layers of transcriptional regulation. The promoters of the telomeric members of the gene family have a strong Gal4 binding site, suggesting they may be coordinately regulated by this transcription factor [23]. *TLO* genes are also subject to local, chromatin-mediated positional effects that result in highly variable expression patterns from cell to cell and population to population [24]. This “noisy” expression pattern has been termed Telomere-Adjacent Gene Expression Noise (TAGEN) and results in highly variable patterns of *TLO* expression between individual cells and even between alleles of the same *TLO* gene. Mechanistically, this variation is dependent on telomere position and silencing regulators such as Sir2 [24]. Ectopically expressed genes at subtelomeric regions were also subject to TAGEN. Interestingly, when the *URA3* gene, which can be subjected to both positive and negative selection, was placed adjacent to a *TLO* gene, and high-level or low-level expression was selected, the level of TAGEN was reduced [24]. This illustrates that some selective pressures can influence the natural level of gene expression noise. Furthermore, because their expression is noisy, the range of assembled Mediator complexes containing a given Tlo/Med2 subunit can vary greatly from cell to cell, generating an epigenetic mechanism for phenotypic diversity within an isogenic population of cells.

## Future Directions

**Fig 2:**
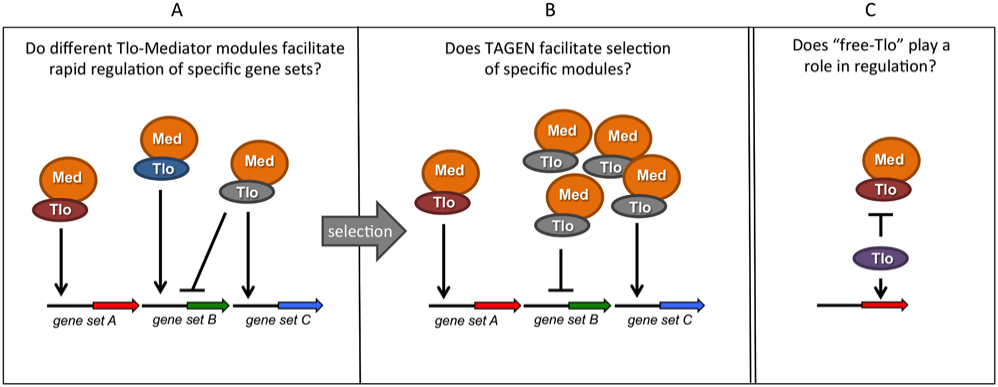
Summary of hypotheses on the possible function(s) of multiple *TLO* genes in *C*. *albicans*. Tlo proteins are subunits of the tail module of the Mediator complex (Med). (A) Different Tlo proteins could facilitate high-affinity interaction of Mediator with specific promoters or transcription factors, facilitating rapid or high level transcriptional responses. (B) As a consequence of telomere-associated gene expression noise (TAGEN) exhibited by *TLO* genes, adaptive pressure may select populations of cells expressing specific Tlos. (C) Excess, non-Mediator—associated “free Tlo” may also exhibit regulatory functions, either independently of Mediator or perhaps in an antagonisitic fashion.

Many questions about *TLO* gene function remained unanswered (Fig. 2). One key piece of information currently absent from our knowledge is whether the *C*. *albicans* Tlo proteins exhibit functional diversity. Heterologous expression of the various *C*. *albicans TLO* genes in *C*. *dubliniensis* may provide clues about their specific regulatory functions. These data may support the hypothesis that differential expression of specific Tlos could provide a selective advantage in specific environments. In support of these heterologous expression experiments, in vitro and in vivo selection experiments with *C*. *albicans* may enable us to generate strains of *C*. *albicans* with a fitness advantage conferred by expression of specific *TLO*s. These data may enable us to determine whether the *TLO* expansion in *C*. *albicans* contributes to its greater pathogenicity relative to its *TLO-*deficient relatives, *C*. *tropicalis* and *C*. *dubliniensis*.
